# Endocrine and Metabolic Effects of Nutrition in Children and Adolescents

**DOI:** 10.3390/nu17040707

**Published:** 2025-02-17

**Authors:** Éva Erhardt, Dénes Molnár

**Affiliations:** 1 Department of Pediatrics, Medical School, University of Pécs, H-7266 Pécs, Hungary; erhardt.eva@pte.hu; 2 National Laboratory for Human Reproduction RRF-2.3.1-21-2022-00012, Szentágothai Research Centre, University of Pécs, H-7266 Pécs, Hungary

Child health is a cornerstone of social well-being, and nutrition plays a crucial role in shaping the growth and development of young individuals. Beyond caloric intake, nutrition influences the complex hormonal and metabolic pathways that govern physical, cognitive, and emotional development. Balance is vital, as both undernutrition and overnutrition disrupt this delicate equilibrium, leading to immediate and long-term consequences. At present, many countries in the world suffer from a double burden of malnutrition, which consists of both undernutrition and overweight and obesity, as well as diet-related noncommunicable diseases [1,2]. In spite of the exponential growth of our knowledge about the relationship between nutrition and health or ill-health, many gaps remain, especially concerning the mechanisms that explain how the different nutrients affect the human body. The main goal of this Special Issue was to address some of these knowledge gaps.

It is not surprising that five of the six papers published in this Special Issue are related to childhood obesity, since childhood obesity has been recognized by the World Health Organization as the most important public health issue in developed countries [3]. During a very short period of time (approximately four decades), obesity has become a global epidemic and an urgent health and economic burden due to its impact on public health and on society as a whole [4]. Obesity is a complex multifactorial disease defined by excessive adiposity and is linked to an increased risk for many noncommunicable diseases.

Two papers are original studies. One explorative study investigated the role of the tryptophan–kynurenine pathway in pediatric obesity and its relationship with obesity-associated metabolic comorbidities [5]. There is no doubt that this paper directs our attention to a rarely investigated pathway in childhood obesity. The other original paper investigated the impact of pandemic restrictions in children with endocrine diseases [6]. The main finding of the study is that the pandemic restrictions decreased physical activity and increased sleeping time leading to an increase in BMI. The study emphasizes that particular attention should be paid to the maintenance of a healthy lifestyle during situations like the recent pandemic.

Three reviews in the Special Issue target different areas of childhood obesity and provide up-to-date information. Prader–Willi syndrome (PWS) was the first genetic syndrome attributed to genomic imprinting. PWS is a neurodevelopmental genetic condition due to paternal loss of imprinted genes on chromosome 15 [7]. PWS is an exceptionally complex disorder with multifaceted clinical challenges ranging from poor appetite and failure to thrive to hyperphagia and extreme obesity. The review by Erhardt and Molnar [8] provides new information about the complex and still unresolved treatment of the syndrome.

Promoting fruit and vegetable consumption in children is a key strategy for addressing childhood nutrition and preventing diet-related health issues. Advertising can play a significant role in shaping children’s food choices, both positively and negatively.

The narrative review by Folkvord et al. [9] gives an overview of how food promotional efforts might be a useful tool to increase the attractiveness of fruit and vegetables in order to change our environment from an obesogenic to a healthogenic one.

The prevalence of hypertension in childhood and especially during adolescence has increased in recent decades [10]. Obesity, sodium homeostasis, and arterial hypertension in children and adolescents are interrelated issues. The interplay between obesity, sodium homeostasis, and hypertension is a critical area of concern for public health. The narrative review by Wojcik and Koziol-Kozakowska [11] demonstrated the pathological mechanisms of hypertension development in obese individuals, both the traditional and new concepts (involving the role of fat tissue).

The primary focus for insulin dosing in type 1 diabetes mellitus is carbohydrate intake, as carbohydrates have the most significant and immediate impact on blood glucose. However, monitoring protein and fat intake is also important for optimal control. A thorough systematic review [12] in the present Special Issue clearly demonstrates that the amount of protein and fat consumption also significantly influences the postprandial glucose levels. In spite of this scientific evidence, current recommendations are still solely based on carbohydrate content. The authors state that “high fat and/or high protein meals require more bolus insulin than low fat/low protein meals with identical amount of carbohydrates”, and therefore, new recommendations and methods for calculating the amount of bolus insulin are required.

In conclusion, the papers featured in this Special Issue shed light on various aspects of childhood obesity, hypertension and type 1 diabetes mellitus. They explored potential new mechanisms and raise important scientific questions for future research.

As guest editors of this research topic, we are delighted to present high-quality contributions embracing many aspects of nutrition in this Special Issue and we do hope that the published papers will be useful for people working in basic and clinical sciences and also for those interested in public health.
